# Facial nerve grading after parotidectomy

**DOI:** 10.1007/s00405-014-3196-y

**Published:** 2014-07-09

**Authors:** Dominik Stodulski, Andrzej Skorek, Bogusław Mikaszewski, Piotr Wiśniewski, Czesław Stankiewicz

**Affiliations:** 1 Department of Otolaryngology, Medical University of Gdańsk, ul. Smoluchowskiego 17, 80-214 Gdańsk, Poland; 2 Department of Endocrinology and Internal Medicine, Medical University of Gdańsk, Gdańsk, Poland

**Keywords:** Parotid surgery, Parotidectomy, Complications, Facial nerve grading, Scale

## Abstract

Dysfunction of the facial nerve is a common complication of parotidectomy. The functional deficit may be total or partial, and may include all or a single branch of the nerve. Despite a wide variety of the facial nerve grading systems, most of them have a limited utility in patients after parotidectomy. Therefore, existing scales assessing facial nerve function are compared to describe facial nerve outcomes after parotidectomy. The regional House–Brackmann, Sydney, and Yanagihara classification systems were utilized. The post-parotidectomy facial nerve grading system (PPFNGS) was created based on these three grading systems and also used for this study. The facial nerve function was assessed and recorded on the first postoperative day following conservative parotidectomy in 200 patients using all 4 scales by 3 otolaryngologists. The validity of the PPFNGS and existing facial nerve grading systems was examined by assessment of interrater agreement, intraclass correlation coefficient, internal consistency and construct validity. A deficit in the facial nerve function was found in 54 patients (27 %). Although results were consistent in all tested scales, the PPFNGS had a higher interrater agreement than the other three scales. PPFNGS is a new grading system designed for assessing the facial nerve function after parotidectomy in a quantitative and qualitative way and has a higher interrater agreement than other scales used to examine function of the 7th nerve.

**Electronic supplementary material:**

The online version of this article (doi:10.1007/s00405-014-3196-y) contains supplementary material, which is available to authorized users.

## Introduction

Parotidectomy is a well recognized and common surgical procedure used to treat tumors in the parotid gland. Dysfunction of the facial nerve is a common and typical complication of this surgical technique even though its anatomic continuity is preserved [1]. The deficit of the nerve function may be total (paralysis) or partial (paresis), and from injury to the main trunk or only the individual branches. According to data from the world literature, postoperative transient facial nerve dysfunction occurs up to 46.1 % of cases, permanent damage is much less common, occurring 1.9–3.9 % [2, 3]. Dysfunction of the 7th nerve occurs most frequently to the marginal mandibular branch—64.1 %, followed by buccal—20.5 %, zygomatic and temporal branches at 7.7 % [3]. Apart from the cosmetic defect (facial contortion), the most troublesome for the patient are paresis of the zygomatic branch (inability to close the eye completely and corneal drying) and the marginal mandibular branch (difficulty in eating, drinking, and speaking). Paresis or paralysis of the cervical branch is negligible [4]. The results presented in the literature on the facial nerve dysfunction are unclear, usually given in the terms of “paresis” or “paralysis”, without specifying the degree of its severity. Only a few publications describe the branches involved. Therefore, the authors decided to review the existing scales assessing function of the facial nerve in relation to their use in patients after parotidectomy.

## Methods

### Facial nerve grading systems review

The following scales were analyzed for the assessment of the facial nerve function after parotidectomy: Adour and Swanson System, Burres–Fisch Linear Measurement Index (BFLMI), the Nottingham System, detailed evaluation of facial symmetry (DEFS), global and regional House–Brackmann, Sunnybrook, Sydney, Yanagihara facial nerve grading systems [5–13]. Because the function of the entire facial nerve and its individual branches is desired (quantitative and qualitative), the authors rejected the scales showing only the global (quantitative) function of the facial nerve which are the Adour and Swanson System, BFLMI, DEFS, global House–Brackmann, Sunnybrook and the Nottingham System. The three grading systems were chosen, regional House–Brackmann (RHBS), Sydney (SS) and global Yanagihara scale (YNFGS), which allow individual assessment of separated facial regions. The Sydney facial grading system evaluates function of the five facial nerve branches (including the cervical branch) for the targeted movements of the facial muscles groups supplied by these branches, giving each from 0 to 3 points, and the result is presented with the points (0–3) granted for synkineses [12]. The regional modification to the House–Brackmann scale assesses four facial regions at rest and during movements (forehead, eye, midface, and mouth), awarding from 1 to 6 points (1, normal; 6, paralysis) [10]. As used in Japan, the Yanagihara grading system investigates different facial muscles at rest and during 9 separate actions, giving points from 0 to 4. The total score ranged from 0 (complete paralysis) to 40 (full function). Most of the functions being examined concerns the eye (4) and mouth (3), which reflect isolated paresis, but this scale does not provide a qualitative deficit of individual branches of the facial nerve [13]. Regional House–Brackmann, Sydney and Yanagihara facial nerve grading systems were presented in electronic supplementary material.

### Creation of the own facial nerve grading system

Based on these scales, the authors decided to create their own system taking into account specifics of the facial nerve dysfunction in patients after parotidectomy. This was named the post-parotidectomy facial nerve grading system (PPFNGS). This scale examines the function of four branches of the facial nerve and it was based on the evaluation of facial symmetry at rest, during spontaneous (blinking, talking, smiling) and voluntary movements of the facial muscles (forehead, eye, cheek, mouth) by performing the following steps: wrinkling the forehead and raising eyebrows (temporal branch), closing the eyes (zygomatic branch), raising the cheeks and wrinkling the nose (buccal branch), and whistling and showing the teeth (buccal branch—upper part and marginal mandibular branch—lower part of the mouth). Activity was evaluated by giving to the each branch of the facial nerve from 0 to 4 points. Full symmetry at rest with full movements—4 points (complete function), symmetry at rest with a slight asymmetry with complete movements—3 points (slight paresis), symmetry at rest with a clear asymmetry with movements—2 points (pronounced paresis), asymmetry at rest with a trace of movement—1 point (profound paresis), and asymmetry in the rest of the complete lack of mobility—0 points (paralysis of all branches).

Slight paresis represents normal symmetry at rest, but only a slight asymmetry of facial function with motion. This form of paresis does not interfere with complete eye closure, puckering of the lips to whistle or smile, or raising of the eyebrows. Pronounced paresis represents normal symmetry at rest, but obvious asymmetry with motion that also interferes with function, such as inability to close the eye completely.

To assess the qualitative presentation of facial paresis, a score from 0 to 4 was given to measure the function of each facial nerve branch (T, temporal; Z, zygomatic; B, buccal; M, marginal mandibular). Tables 1 and 2 show the principles of scoring, evaluating and recording of the facial nerve function after parotidectomy.

**Table 1:** Scoring rules of the facial nerve function after parotidectomy (PPFNGS)

Degree	Description	Points
Complete function	Symmetry at rest Symmetry at full range of movements	4
Slight paresis	Symmetry at rest Slight asymmetry at full range of movements	3
Pronounced paresis	Symmetry at rest Movement disorders with clear asymmetry	2
Profound paresis	Asymmetry at rest Slight of the muscle movements	1
Paralysis	Asymmetry at rest Lack of movements	0

**Table 2:** Assessment and recording of facial nerve function after parotidectomy (PPFNGS)

7th nerve branch	Symmetry at rest and spontaneous movements	Assessed function	Points
Temporal (T)	Forehead wrinkles Eyebrows level	Forehead wrinkle Eyebrows raise	0–4
Zygomatic (Z)	Blinking	Eye closure	0–4
Buccal (B)	Nasolabial folds symmetry	Cheeks raise Nose wrinkle	0–4
Marginal mandibular (M)	Speech^a^ Smile^a^ Mouth corner symmetry	Whistle^a^ Showing teeth (grin)^a^	0–4
Whole nerve VII			0–16 (Tx, Zx, Bx, Mx)

^a^Also for buccal branch

For example, full function of all four branches is scored as 16 (T4, Z4, B4, M4). Slight paresis (3 points) of only marginal mandibular branch is scored as 15 (T4, Z4, B4, M3). Profound paresis (1 point) of the temporal branch and pronounced paresis (2 points) of the zygomatic branch is scored as 11 (T1, Z2, B4, M4). Paralysis of all branches (0 points) is given a score 0 (T0, Z0, B0, M0). The average score for global facial assessment in the sample group is 10.5, while the mean score when assessing only cases with facial nerve dysfunction is 8.6 (T1.6, Z2.0, B2.6, M2.3).

In our new scale the synkinesis and mass contracture were not taken into consideration; however, there is a potential possibility to present these abnormalities by adding the letter S while recording the nerve function, for example 12S (T2S, Z2S, B3, M3).

### Clinical test of four facial nerve grading systems

The cross-sectional study with planned data collection was conducted between 2010 and 2012 in the Department of Otolaryngology, Medical University of Gdańsk. Facial nerve function was assessed independently by three otolaryngologists—head and neck surgeons in 200 patients (110 women and 90 men, age from 20 to 88 years, the mean age was 53.4), during the first day after conservative parotidectomy. Function of the facial nerve was measured using PPFNGS and with the three existing systems (RHBS, SS, YFNGS).

### Statistical analysis of tested facial nerve grading systems

Validity of the new and the selected existing functional facial nerve grading systems was examined by assessment of interrater agreement, intraclass correlation coefficient, internal consistency and construct validity. Interrater agreement was assessed using the weighted kappa-statistic for three raters. We used a mixed ANOVA model to estimate intraclass correlation coefficient, i.e. the proportion of the between-subject variance to the total variance. The remaining part of the total variance reflects the inter-observer variance. Agreement between the new instrument and the existing ones was assessed using Bland–Altman method with regression adjustment for the proportional bias. Correlations between PPFNGS and the other scales were assessed using Spearman rank correlation. Statistical analysis was carried out using STATA 13.0 statistical package software (StataCorp, TX, USA).

## Results

**Fig. 1:**
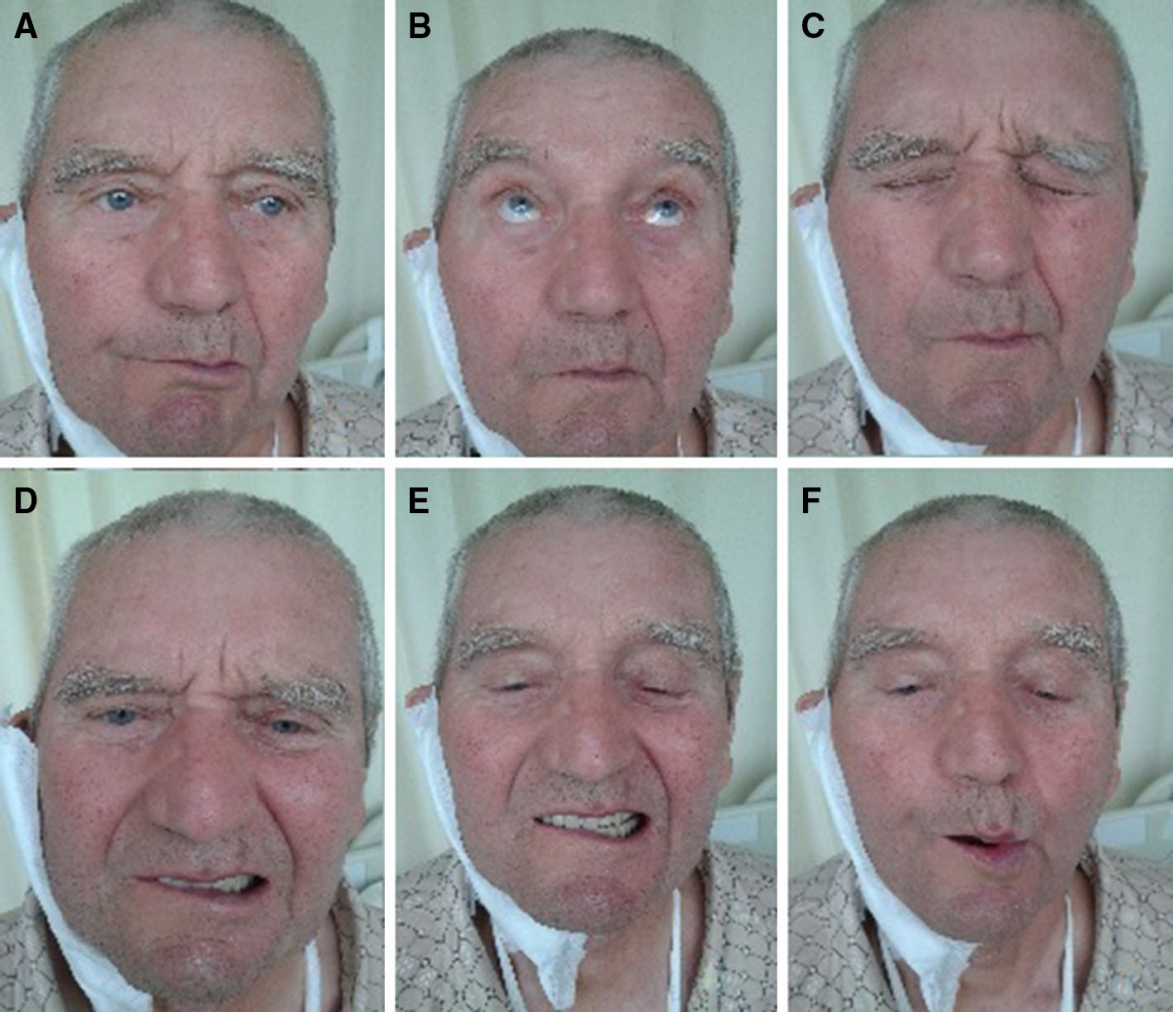
Patient with right-sided post-parotidectomy facial nerve paresis: at rest (**a**), during raising the eyebrows (**b**), closing the eyes (**c**), wrinkling the nose (**d**), showing the teeth (**e**), and whistling (**f**)

Mean duration of the facial nerve examination was approximately 3 min. In the postoperative assessment of the facial nerve, a function deficit was found in 54 patients (27 %). The marginal mandibular branch was involved in 29 patients, the temporal in 4 patients, temporal and zygomatic in 4 other patients, and all branches in 17 patients. In the remaining 146 patients who underwent surgery, according to all the examining specialists, the facial nerve function was unaffected. Records of the analyzed group of patients in four tested systems by a single observer are shown in Table 3. Figure 1 presents the patient with post-parotidectomy facial nerve paresis at rest and during voluntary movements. Table 4 shows the recorded function of the facial nerve of the patient from the figure in four tested systems assessed by one observer.

**Table 3:** The recorded function of the facial nerve in the investigated group in four tested systems assessed by one observer

7th nerve function	*n*	PPFNGS	SS	RHBS	YS
Norma	146	16 (T4; Z4; B4; M4)	15	4	40
M branch deficit	29	13.73 (T4; Z4; B4; M2.27)	13.2	5.63	34.2
T branch deficit	4	14 (T2; Z4; B4; M4)	13.5	5.5	32
T and Z branches deficit	4	13 (T2.5; Z2.5; B4; M4)	12.5	6.5	27
All branches deficit	17	9.3 (T2.3; Z2.3; B2.46; M2.3)	8.64	8.34	24.7
Average paresis	54	12.6 (T3.23; Z3.31; B3.44; M2.6)	11.5	11.8	30.8
Average in whole group	200	15	14.1	5.19	37.5

*M* marginal mandibular, *T* temporal, *Z* zygomatic, *PPFNGS* post-parotidectomy facial nerve grading system, *RHBS* Regional House–Brackmann system, *SS* Sydney system, *YS* Yanagihara system

**Table 4:** The recorded function of the facial nerve of the patient from the figure in four tested systems assessed by one observer

System	Score	Total paralysis	Normal function
Post-parotidectomy facial nerve grading	12 (T4; Z3; B4; M1)	0	16
Regional House–Brackmann	8	24	4
Sydney	12	0	15
Yanagihara	19	0	40

*T* temporal, *Z* zygomatic, *B* buccal, *M* marginal mandibular

### Statistic analysis

*Interrater agreement*. The kappa value for PPFNGS was 0.935 indicating almost perfect interrater agreement and was markedly higher compared to the other systems: Yanagihara 0.765, RHB 0.749, Sydney 0.645. The values for PPFNGS were higher than for its comparatives with respect to the individual branches. The minimal kappa for the proposed grading system was 0.94 indicating its superior interrater reliability in functional assessment of each of the peripheral facial nerve branches. Values of intraclass correlation coefficient (ICC) for the tested systems ranged 0.971–0.997 (PPFN 0.997, Yanagihara 0.996, RHB 0.994, Sydney 0.971). This indicates that nearly all of the total variability in patients scores resulted from between-subject differences and only 0.2–2.8 % of the variability was due to inter-observer differences.

*Agreement*. PPFNGS showed substantial overall agreement with the examined grading systems. The highest observed Bland–Altman agreement between PPFNGS and the 3 tested grading systems (as proportion of results outside the 95 % limits of agreement) was for RHB 1.42 %, then for Yanagihara 4.65 % and the lowest for Sydney system (7.38 %).

*Correlation*. The results of PPFNGS were highly correlated with the results of other scales. All of the correlation coefficients exceeded 0.9 (PPFNG vs. Yanagihara – 0.982; PPFNG vs. Sydney 0.961, PPFNG vs. RHB 0.929).

## Discussion

Adequate assessment of the facial nerve function after parotidectomy requires attention to the individual facial nerve branch deficits and their degree of function. The most appropriate scale should be able to allow evaluation of the degree of damage to the individual branches of the facial nerve in a quick and reproducible manner that is not cumbersome. The existing grading systems to measure the function of the facial nerve can be described as global and regional, as well as subjective and objective. The global scales give a rating of the overall facial nerve function, which is most applicable in lesions or injuries to the trunk of the facial nerve, as in Bell’s palsy, herpes zoster, pyramid fractures, after surgery of the tumors of the ponto-cerebellar angle or middle ear. “Trunk” paresis after parotidectomy also may occur, but it is much less frequent and may result either from damage to the nerve trunk or its branches (in different sites).

The most commonly used global House–Brackmann grading system (GHB) was developed to assess the paresis of the facial nerve after surgery of the ponto-cerebellar angle tumors [9]. Studies that use the House–Brackmann scale to describe parotidectomy injuries to the facial nerve may overlook paresis of isolated branches of the nerve. Therefore, the true incidence of the facial nerve paresis following parotidectomy in the literature is questioned given the inadequacies of the grading scales.

Objective systems are based on measurements of the distance between certain points on photographs of the face (BFLMI and its modifications—the Nottingham System), but these systems are time-consuming, complex, and not amenable to simple bedside examination [6, 7]. Croxson et al. [14] compared a subjective scale (GHB) to an objective one (BFMLI) and found a high concordance between them. They could not prove whether one scale was superior to the other, because the former is a subjective and qualitative scale and the latter is an objective and quantitative scale. Although these scales attempt to improve the accurate description of damage to the facial nerve function, a universal scale is not agreed upon.

Currently, there is a tendency to create automated functional assessment of the facial nerve. However, they require special software, are time-consuming and based mainly on evaluation of the certain landmarks and distances on the face on pictures/facograms, etc. The disadvantages of this method are that it requires a normal side for comparison and standardization. The presence of some individual differences between left and right side of the face, for example strabismus, artificial eye or post-traumatic deformity, might also lead to difficulties in facial nerve grading [15].

Because objective scales are time-consuming and require complicated measurements, subjective scales are more commonly used at the bedside even though they are more prone to variability between raters.

The five regions of the face and neck innervated by the 7th cranial nerve (forehead, eye, cheek, mouth, neck) and the degree of impairment of each region should be included in any new grading system. The more areas surveyed, the more detailed the scale becomes. However, it should be noted that the most important innervation deficits for patients involve the eye and mouth (not the forehead, cheek, or neck). Only the Sydney scale assesses the cervical branch; however, inclusion of the cervical branch may obscure the impact of injury to more important branches of the nerve in their total assessment. The Sydney and DEFS scales only describe two degrees of paresis, which may be less accurate than other scales such as the regional House–Backmann scale that has four levels of paresis. However, scales such as the House–Brackmann scale that have a high range in scores may make it more difficult to compare patients [8, 10, 12]. Rickenmann et al. [16] compared the GHB scale with DEFS and found that the simpler assessment systems show greater compatibility between observers; however, the precision of paresis assessment is affected by the degrees of paresis in the grading system. The five-step rating system that incorporates three degrees of paresis on the top of complete paralysis and full function outcomes seems to be the best compromise between accuracy and low complexity. A “pronounced paresis” is located in the middle of the scale and it is a reference point for other grades of paresis (slight versus profound) and simplifies facial function evaluation. Another problem is the smaller range of activities of the cheeks and forehead muscles, since their participation in spontaneous movements is less clear and the range of targeted motion harder to quantify. As already mentioned, the deficit of their activities is also less important, and perhaps for this area it would be beneficial to use only 2 degrees of paresis. The exact determination of facial nerve function is sometimes very difficult. The authors feel that in the setting of uncertainty, the score should be upscaled to indicate the worst-case scenario.

The iatrogenic facial nerve dysfunction (one/several branches, or trunk) after parotidectomy has a different mechanism (pulling, pressure, the use of electrocautery) than in Bell’s palsy or Ramsay–Hunt Syndrome, this is why the synkineses associated with abnormal axon regeneration are not present. Thus, in the presented scale, the authors did not include synkineses (rated in Sunnybrook and Sydney scales) [11, 12].

As demonstrated by the statistical analysis of the facial nerve function, the results were consistent and highly correlated in all tested scales. PPFNGS proved to have somewhat higher interrater agreement compared with the regional House–Brackmann, Sydney and Yanagihara systems. The advantages of new scale is also its possibility of a precise description of the facial nerve function in an individual patient, as well as the presentation of mean values and the degree of paresis of the nerve branches in the entire group of patients. The disadvantage of the scale is its subjectivity, the difficulty in grading the temporal and buccal branches paresis and (forehead and cheek movements) and the presence of a fraction of the average values for a group of patients with paresis.

The postoperative facial nerve dysfunction is not only a cosmetic problem, but a functional problem as well. Depending on locations of the injuries to the nerve trunk or branches, important functions such as facial expression, eye protection, eating, drinking, and speech can be affected. This is why it is necessary to use an appropriate scale to measure all aspects of the facial nerve function. Although this scale can be used to compare outcomes (complications), it may also be applicable in legal proceedings, insurance (compensations), and rehabilitation outcomes.

## Conclusions

Post-parotidectomy facial nerve grading system is a new grading system designed for assessing the facial nerve function after parotidectomy. The PPFNGS is simple to use at the bedside, assesses all clinically important motor branches of the facial nerve, and has a higher interrater agreement than other scales used to examine function of the 7th cranial nerve.

## Electronic supplementary material

Below is the link to the electronic supplementary material.

Supplementary material 1 (PDF 89 kb)
