# Supplementary material for: Facial nerve grading after parotidectomy
Source: Eur Arch Otorhinolaryngol. 2014 Jul 9;272(9):2445–50. doi: 10.1007/s00405-014-3196-y (PMC4526578; doi:10.1007/s00405-014-3196-y)
Supplement: Supplementary file 1 — Supplementary material 1 (PDF 89 kb) [file 405_2014_3196_MOESM1_ESM.pdf]

**European Archives of Oto-Rhino-Laryngology and Head & Neck**

# Facial nerve grading after parotidectomy

Dominik Stodulski, Andrzej Skorek, Bogusław Mikaszewski,* Piotr Wiśniewski, Czesław Stankiewicz

Department of Otolaryngology, Medical University of Gdańsk, Poland

*Department of Endocrinology and Internal Medicine, Medical University of Gdańsk, Poland

Corresponding author: e-mail: dstodulski@gumed.edu.pl

## Sydney facial grading system

Voluntary Movement of the 5 Branches of Facial Nerve

| | |
|---|---|
| Temporal (T): | Forehead raise/frown |
| Zygomatic (Z): | Eye closure |
| Buccal (B): | Nose wrinkle, pout and smile – upper mouth and cheek |
| Marginal mandibular (M): | Lips pulled down- Chin region |
| Cervical (P): Platysma | |

| | |
|---|---|
| Normal facial movement | 3/3 |
| Moderate amount of facial movement | 2/3 |
| Small amout of facial movement present | 1/3 |
| No facial movement | 0/3 |

| | |
|---|---|
| Synkinesis of Overall Face | |
| Severe synkinesis | 3/3 |
| Moderate synkinesis | 2/3 |
| Mild synkinesis | 1/3 |
| No synkinesis | 0/3 |

| Score | 0 | 1 | 2 | 3 |
|---|---|---|---|---|
| T | | | | |
| Z | | | | |
| B | | | | |
| M | | | | |
| C | | | | |
| Synkinesis | | | | |

**Regional House-Brackmann (RHB) facial nerve grading system**

| | Grade | Description |
|---|---|---|
| Forehead | 1 | Normal forehead movement |
| | 2 | Slight weakness in forehead movement |
| | 3 | Obvious but not disfiguring asymmetry with motion, symmetric at rest |
| | 4 | Obvious weakness of disfiguring asymmetry with motion, symmetric at rest |
| | 5 | Barely perceptible motion in forehead, asymmetric at rest |
| | 6 | No movement |
| Eye | 1 | Normal eye closure |
| | 2 | Mild weakness in eye closure |
| | 3 | Obvious weakness but able to close eyes |
| | 4 | Unable to close eye with maximal effort |
| | 5 | Barely perceptible eyelid movement |
| | 6 | No movement |
| Midface | 1 | Normal midface movement |
| | 2 | Slight weakness in midface movement |
| | 3 | Obvious but not disfiguring weakness, symmetric at rest |
| | 4 | Obvious weakness and disfiguring asymmetry with motion, symmetric at rest |
| | 5 | Barely perceptible motion in midface, asymmetric at rest |
| | 6 | No movement |
| Mouth | 1 | Normal corner of mouth movement |
| | 2 | Slight weakness of corner of mouth movement |
| | 3 | Obvious but not disfiguring weakness, symmetric at rest |
| | 4 | Obvious weakness and disfiguring asymmetry with motion, symmetric at rest |
| | 5 | Barely perceptible corner of mouth movement, asymmetric at rest |
| | 6 | No movement |
| Synkinesis | None | None |
| | Mild | Obvious but not disfiguring |
| | Severe | Disfiguring or interferes with function |

**Yanagihara five-point facial nerve grading system (0 to 40)**

| | Paralysis | | | | Normal |
|---|---|---|---|---|---|
| | total | severe | moderate | slight | |
| At rest | 0 | 1 | 2 | 3 | 4 |
| Wrinkle forehead | 0 | 1 | 2 | 3 | 4 |
| Blink | 0 | 1 | 2 | 3 | 4 |
| Slight closure of eye | 0 | 1 | 2 | 3 | 4 |
| Tight closure of eye | 0 | 1 | 2 | 3 | 4 |
| Closure of eye on the involved side only | 0 | 1 | 2 | 3 | 4 |
| Wrinkle nose | 0 | 1 | 2 | 3 | 4 |
| Whistle | 0 | 1 | 2 | 3 | 4 |
| Grin | 0 | 1 | 2 | 3 | 4 |
| Depress lower lip | 0 | 1 | 2 | 3 | 4 |
